# A case report: The first show phenomenon in the treatment of spinal cord injury with Regentime procedure using autologous bone marrow‐derived stem cells

**DOI:** 10.1002/ccr3.7568

**Published:** 2023-07-02

**Authors:** Rita T. Boulos, Lea I. Nemer, Vanessa J. Mansour, Cynthia F. Najjoum, Elsa A. Asmar, Nassim H. Abi Chahine

**Affiliations:** ^1^ Stem Cell Transplantation/Neurology ACE Cells Lab Limited Beirut Lebanon; ^2^ Stem Cell Transplantation/Molecular Biology ACE Cells Lab Limited Beirut Lebanon; ^3^ Stem Cell Transplantation/Infectious Diseases/Immunology ACE Cells Lab Limited Beirut Lebanon; ^4^ Stem Cell Transplantation/Functional Genomics/Proteomics ACE Cells Lab Limited Beirut Lebanon; ^5^ Stem Cell Transplantation/Neurological Surgery ACE Cells Lab Limited Beirut Lebanon

**Keywords:** autologous bone marrow‐derived mononuclear stem cells, first show phenomenon, Regentime therapy, spinal cord injury, stem cell therapy

## Abstract

**Key Clinical Message:**

Promising outcomes are shown in this case report using the Regentime procedure and autologous stem cells to treat spinal cord injury. The observed “First Show Phenomenon” provides valuable insights into the therapy's potential for spinal cord injury.

**Abstract:**

This case report demonstrates “the first show phenomenon” following Regentime stem cell therapy applied to a spinal cord injury patient. A 40‐year‐old gentleman sustained a ballistic injury at the level of T9, resulting in complete bilateral motor and sensory loss from T9 and below. He was treated with autologous bone marrow‐derived mononuclear stem cells injected into his spinal canal 2.5 years after his injury. Follow‐up during the first‐week posttransplantation showed early symptom improvement termed “the first show phenomenon.” He regained sensation to light touch in his lower limbs by the end of week 1 and reported no serious implications or complications.

## 1 INTRODUCTION

Spinal cord injury (SCI) is a critical health condition that can result in substantial morbidity and long‐term disability. The disruption of the electrical influx running through the spinal cord leads to sensory‐motor loss of function below the injury level.^1^ The leading causes of SCI include motor vehicle accidents, violence (especially gunshot wounds), sports accidents, and falls among others.^2^ The pathophysiology of SCI consists of primary and secondary phases of injury. The primary phase relates to the early stage immediately after the injury and includes neural parenchyma destruction, axonal network disruption, glial membrane disruption, and hemorrhage. The secondary phase follows and is manifested by early vascular damage, free radical formation, inflammation, necrosis, neuronal apoptosis, Wallerian degeneration, axonal demyelination and remodeling, and late glial scar formation and maturation.^3^

Despite significant advances in new therapeutic and rehabilitation approaches, there is no treatment for SCI yet. However, several clinical trials were conducted to assess the long‐term therapeutic efficacy of bone marrow‐derived stem cell transplantation on SCI patients and found promising results.^4^, ^5^ We report the case of a 40‐year‐old gentleman with a past medical history of SCI at the level of T9 two and a half years ago, with an American Spinal Injury Association (ASIA) impairment scale grade A, who underwent the Regentime procedure.^6^ The patient gained lower limb sensation and improved to ASIA impairment scale grade B as early as 1 week post stem cell transplantation, a manifestation that we call “the first show phenomenon.” The major focus of this case report is the role of Regentime therapy in optimizing neuro‐regeneration by administering autologous bone marrow‐derived mononuclear cells (BM‐MNCs).

## 2 CASE PRESENTATION

**FIGURE 1:**
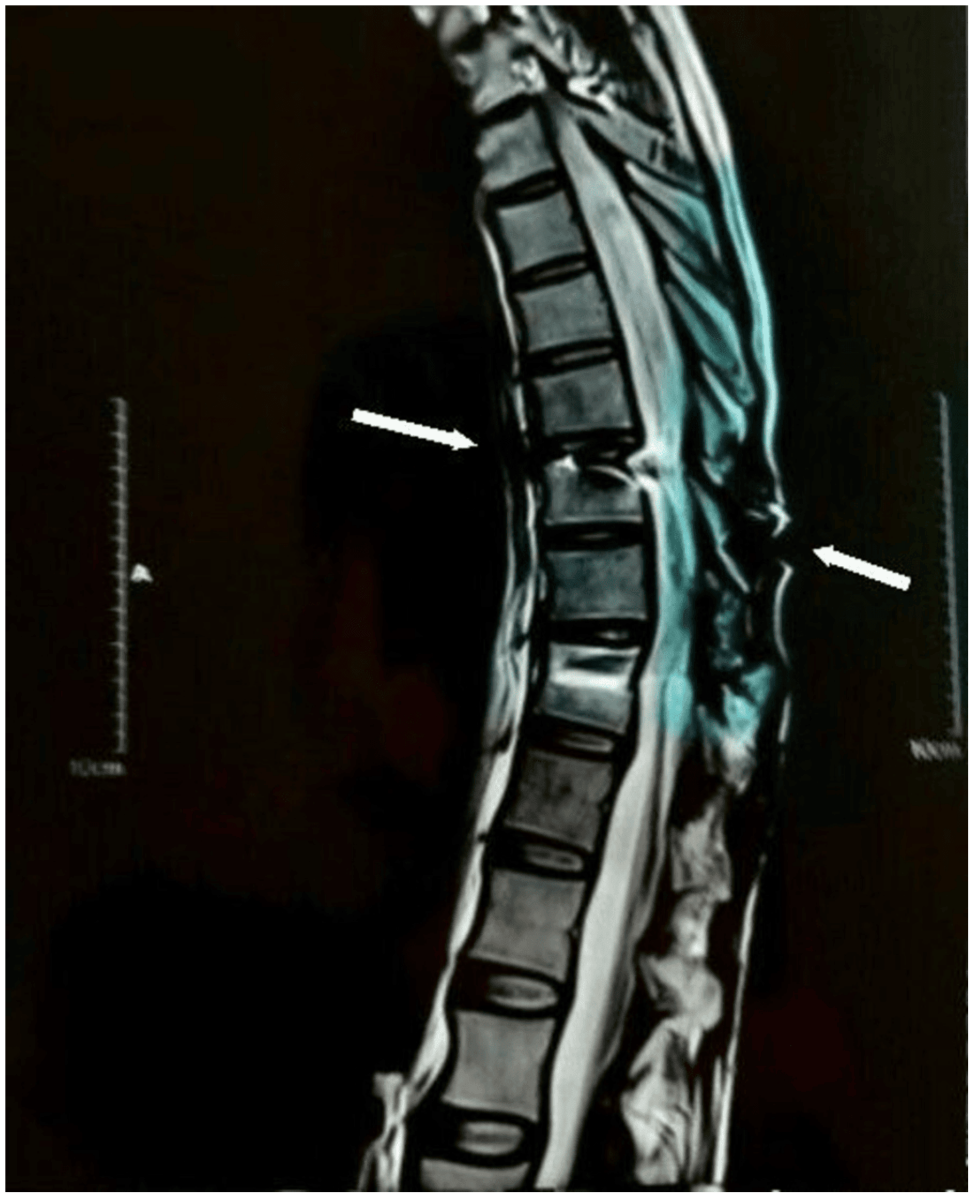
The thoracic injury at the level of T9.

A 40‐year‐old man was presented to our hospital outpatient clinic for a SCI stem cell treatment trial. He had a 2.5 year history of a ballistic injury that resulted in a spinal cord thoracic lesion at the level of T9, for which he underwent posterior decompression and fusion after 24 h (Figure 1). The patient presented with paralysis, loss of sensation in the lower extremities, and loss of bladder and bowel control since the injury. On physical examination, he had a bilateral loss of sensation (pain, temperature, touch, vibration, and proprioception) below the level of injury and a motor power of 0/5 in his lower limbs.

The patient decided to attempt the Regentime Procedure. Before therapy, written consent was obtained from the patient, and a day‐by‐day follow‐up was done during the first weeks. A periodic 1‐month follow‐up was scheduled for later assessment of safety and improvement.

The Regentime Procedure^6^:

1. Pre‐laboratory stage

During the first 2 days, the patient was injected with a total of four doses of 300 mcg of granulocyte colony stimulating factor intramuscularly separated by 12 h each. Eight hours after the last dose, an increase in the white blood cell count was registered 28,000 cells/mL.

2 The bone marrow collection stage

Bone marrow aspiration was done, in which 100 mL were collected from each posterior superior iliac crest, using four aspiration syringes filled with 25,000 units of 10% heparin sodium each. The aspirate was transferred to a transfusion bag after the removal of sodium citrate under sterile conditions. The bag was held at 22°C on a slow three‐dimensional laboratory shaker.

3 Laboratory stage

The cellular buffy coat containing stem cells was collected after centrifugation.

4 Transplantation stage

After 24 h of incubation, the patient received progenitor stem cells via direct injection in the operating room after the hardware removal. Following the surgery, the patient also had an intravenous administration of around 100 million stem cells (1 mL) in 100 mL of normal saline solution.

5 Posttransplantation stage

The patient stayed in his hospital room for several days to monitor vital signs and record any undesirable effects.

## 3 RESULTS

Postoperatively, the patient was recovering smoothly with no significant complaints. He started to regain sensation in his right lower limb upon touch on day 1 after stem cell transplantation. Toward the end of the first week, he was able to detect touch in both of his lower limbs. However, 2–3 weeks after transplantation, the early improvements declined. Moreover, he could not distinguish between two points applied simultaneously to his legs bilaterally, did not feel changes of temperature applied on his lower limbs, and maintained an absent joint position sense bilaterally.

## 4 DISCUSSION

Every year, around 1 million persons suffer from SCI, causing significant morbidity and permanent motor, sensory, and autonomic dysfunction.^1^, ^3^, ^7^ Stem cell therapy for SCI success rate depends on when the injury first occurred and on its grade due to the heterogeneity of SCI pathophysiology. Our patient sustained a SCI that resulted in a total bilateral loss of sensory and motor function below the injury level. He underwent the Regentime procedure via both direct injection in the operating room and intravenous routes 2.5 years after injury. Regentime therapy first show phenomenon was evident during the first days posttransplantation when the patient regained sensation to touch in his lower limbs. However, this phenomenon has shown to be transient few weeks posttransplantation. Noting that he did not complain of any undesirable effects during this follow‐up period. Our results suggest that the Regentime procedure seems to be safe and effective for patients suffering from SCI.

The first cells utilized for treating SCI in both experimental and clinical trials were bone marrow‐derived mesenchymal stem cells (BM‐MSCs).^8^ Motor, sensory, and autonomic functions were partially improved in primary studies involving thoracic spinal cord contusion and BM‐MSCs transplantation.^5^ Similar improvements were recorded in other studies whether BM‐MSCs were given via direct injection into the spinal canal during a surgical act,^9^ intrathecally,^10^ or intravenously.^11^ Also, stem cell treatment was shown to result in improvement in the ASIA score and recovery of motor‐evoked potentials and somatosensory evoked potentials in a trial conducted on 20 adults with chronic SCI. Autologous stem cell therapy administered intrathecally was shown to participate in neuro‐restoration via several pathways as suggested by the nonidentical effects recorded on different neurophysiological tests.^12^ Concerning safety, phases I/II clinical trials using autologous BM‐MSCs in patients with acute, subacute, and chronic SCI showed no long‐term side effects and seemed to be safe.^13^

Spinal cord injury can be caused by various conditions, possibly resulting in permanent impairment. On the other hand, MSCs and their associated cellular products are readily available therapeutic tools for regenerative medicine, including central nervous system diseases and conditions. The ability of autologous BM‐MSCs to release a range of antiapoptotic, neurotrophic, and anti‐inflammatory molecules is thought to be the cause of their therapeutic effects.^13^ These cells can differentiate into neural cells, express neuronal markers, and secrete neuroprotective growth factors that play a key role in neural restoration and regeneration such as glia‐derived, brain‐derived, nerve, and ciliary neurotrophic growth factors, and neurotropin‐3.^13^, ^14^ On the other hand, cavitation prompted by tissue necrosis, axonal degeneration, and neural cell death is frequently present with damaged neural tissue. This invariably produces scars, which are made up of inflammatory immune cells, fibroblasts, extracellular matrix (ECM) deposits, and astrocytes. MSCs exhibit anti‐inflammatory, antiapoptotic, and ECM modulatory properties, leading to neural rejuvenation.^15^ All these properties suggest that MSCs can be beneficial for spinal cord repair and have provided an outline of strategies that scientists are taking toward rejuvenation and restoration.

This case report shows promising improvement of SCI symptoms in our patient. The Regentime therapy first show phenomenon was manifested through the patient's early regain of sensation to touch progressively during the first week following therapy. However, a longer follow‐up duration is yet to be undergone for further assessment of improvement and any undesirable effects related to stem cell therapy.

## 5 CONCLUSION

Spinal cord injury is a serious public health problem with a high morbidity rate. Stem cell transplantation for SCI treatment has been conducted in several studies showing promising results. Regentime therapy involves administering autologous BM‐MNCs to a target population. The administration of this therapy to our SCI patient demonstrated the “first show phenomenon” where he had improved symptoms as early as during the first few days after therapy.

## AUTHOR CONTRIBUTIONS

**Rita T. Boulos:** Writing – original draft; writing – review and editing. **lea nemer:** Writing – original draft; writing – review and editing. **Vanessa J. Mansour:** Writing – original draft; writing – review and editing. **Cynthia F. Najjoum:** Writing – original draft. **Elsa A. Asmar:** Writing – original draft. **Nassim H. Abi Chahine:** Writing – review and editing.

## FUNDING INFORMATION

No funding declared.

## CONFLICT OF INTEREST STATEMENT

There is no conflict of interest to be declared.

## CONSENT

Written informed consent was obtained from the patient to publish this report in accordance with the journal's patient consent policy.

## Data Availability

The data that support the findings of the study are available upon request, ensuring the anonymity and confidentiality of the study participant. Any personal identifiers or potentially identifying information will be appropriately redacted or anonymized to protect the privacy of our patient.
